# Simultaneous separation of naproxen and 6-O-desmethylnaproxen metabolite in saliva samples by liquid chromatography-tandem mass spectrometry: Pharmacokinetic study of naproxen alone and associated with esomeprazol—Results

**DOI:** 10.1371/journal.pone.0278411

**Published:** 2022-12-01

**Authors:** Gabriela Moraes Oliveira, Thiago José Dionísio, Nelson Leonel Del Hierro Polanco, Viviane Silva Siqueira-Sandrin, Flavio Augusto Cardoso Faria, Carlos Ferreira Santos, Adriana Maria Calvo

**Affiliations:** Biological Sciences, Bauru School of Dentistry, University of São Paulo, Bauru, São Paulo, Brazil; Fisheries and Oceans Canada, CANADA

## Abstract

After performing liquid-liquid extraction with ethyl acetate and HCl, samples from 12 volunteers who performed sequential collections after taking a tablet of naproxen alone (n = 6) or associated with esomeprazole (n = 6) were analyzed in a triple quadrupole mass spectrometer 8040 LC MS/MS Shimadzu. Separation of naproxen and its main metabolite 6-O-desmethylnaproxen was performed in a Shim-Pack XR-ODS 75Lx2.0 column and C18 pre-column at 40°C using a mixture of methanol and ammonium acetate 10 mM (70:30, v/v) with an injection rate of 0.3 ml/min. The total analytical run time for each sample was 5 min. The association of naproxen with esomeprazole take considerably longer time to reach the maximum concentration [T_max_ 0.17 h (interquartile range, 0.13–1.95) for naproxen alone and 13.18*h (interquartile range, 10.12–27.15) for naproxen with esomeprazole, p = 0.002], also to be eliminated [T_1/2_ 0.12 h (interquartile range, 0.09–1.35) for naproxen alone and 9.16*h (interquartile range, 7.16–41.40) for naproxen with esomeprazole, p = 0.002] and lower maximum concentrations (C_max_ 4.6 ± 2.5 ug/mL for naproxen alone and 2.04 ± 0.78* μg/mL, p = 0.038). The association of naproxen with esomeprazole showed increased values of AUC_0-t_ [82.06* h*μg/mL (interquartile range, 51.90–157.00) with esomeprazole and 2.97 h*μg/mL (interquartile range, 1.82–7.84) naproxen alone, p = 0.002] in drug concentrations in relation to the naproxen tablet alone, probably, such differences are due to the delay in the absorption of naproxen when it is associated with the drug proton pump inhibitor, esomeprazole. As well as reduced values of full clearance when naproxen is combined with esomeprazole (0.07* μg/h (interquartile range, 0.005–0.01) with esomeprazole and 7.29 μg/h (interquartile range, 3.17–16.23) in naproxen alone, p = 0.002). Both naproxen and 6-O-desmethylnaproxen in saliva samples can be effectively quantified using LC-MS/MS, this methodology proved to be rapid, sensitive, accurate and selective for each drug and allows for the analysis of their pharmacokinetic parameters, in both situations.

## Results

The complete methodology of this article from the “*Registered Report Research Articles*” modality is described in detail in the article published previously in this journal: “*Simultaneous separation of naproxen and 6-O-desmethylnaproxen metabolite in saliva samples by liquid chromatography-tandem mass spectrometry*: *Pharmacokinetic study of naproxen alone and associated with esomeprazole*. Dionísio TJ et al. Plos One. 2020 Aug 11;15(8):e0236297” [1]. At this point, we will describe the results achieved.

This project was approved by the Research Ethics Committee of the Bauru School of Dentistry/University of São Paulo (CAAE 49806115.0.0000.5417), registered in ClinicalTrials.gov (NCT03092193). All volunteers participating in this research were fully informed about the project’s content and procedures and signed a consent form.

**Fig 1:**
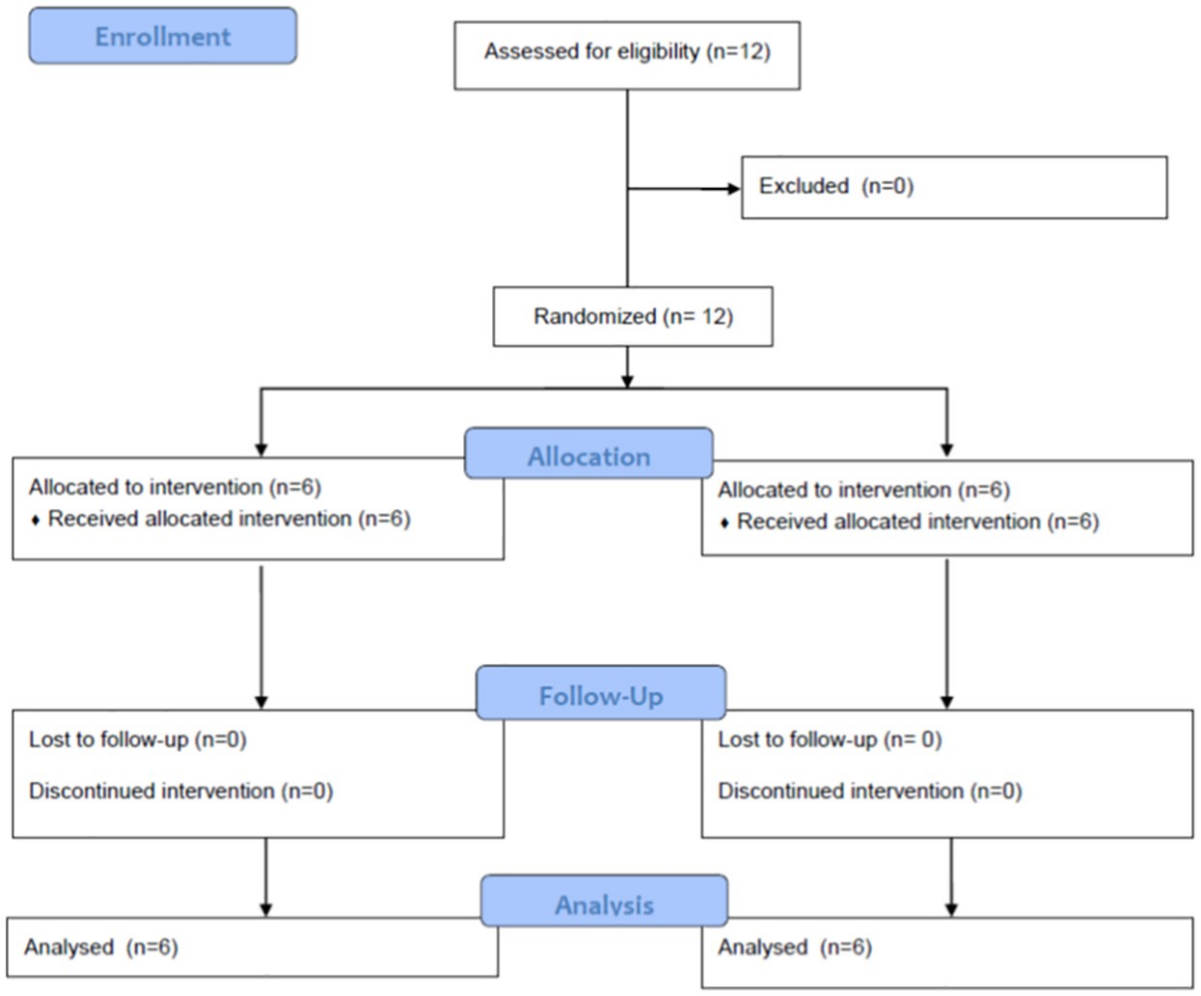
CONSORT flow diagram.

Sequential samples from 12 volunteers were analyzed before and 0.25; 0.5; 0.75; 1; 1.5; 2; 3; 4; 5; 6; 8; 11; 24; 48; 72 and 96h after taking a tablet of naproxen alone (500 mg– 6 volunteers) and naproxen tablets with esomeprazole (500 + 20 mg– 6 volunteers) described in Fig 1. The volunteers were randomly assigned using the Excel software to which group they would be allocated. The principal investigator (AMC) performed the randomization and the data of which sample belonged to which group was only revealed after the end of the experiment. Due to the difficulties imposed by the COVID-19 pandemic, it was not possible to collect samples from the same volunteers for both groups, so the number of samples remained as planned in the methodology already published, there are only different individuals in each group.

### Analysis of data obtained from saliva samples

After analysis on LC MS/MS, the concentrations obtained from saliva samples of naproxen and its main metabolite, 6-O-desmethylnaproxen were transferred to Phoenix WinNonlin Software (version 8.1) for analysis in the non-compartmental model to generate the PK parameters (Table 1).

**Table 1:** Pharmacokinetic parameters analyzed from concentrations resulting from LC MS/MS analysis of saliva samples for naproxen alone or associated with esomeprazole.

Parameters	Description	Methods
Tmax (h) e Cmax (μg/mL)	Time and value of the maximum observed concentration	Graphical Method
AUC_0-t_ (h*μg/mL)	Area under the curve from zero to last quantifiable time	Trapezoid Methods
Vd/f (L)	Apparent volume of distribution	Cl/Kel
Cl/f (L/h)	*Cleareance*	[(Dose)/AUCinf]
K_el_ (1/h)	Elimination constant (ƛz)	Determined by log-linear regression
T1/2 (h)	Drug elimination half-life	T1/2 = Ln(2) / K_el_

Data were descriptively organized and analyzed using Prism8 and Sigmaplot (version 14.0) to create the graphics. Shapiro-Wilk test was performed for normality and lognormality test to compare naproxen alone and naproxen associated with esomeprazole; when equal variances not assumed occurred, Welch’s t test was performed; for data that passed the normality test, the unpaired *t test* was used (mean ± SD) and those that did not pass the normality test underwent the Mann-Whitney test, and results were presented as median (interquartile range).

The linearity was evaluated using a linear mathematical model using the weighted equation 1/χ2. To generate the equation of the lines, at least 5 points of the curve were analyzed, being drawn by the method of least squares, by the Software LabSolutions version 5.87, and the value of r2 (correlation coefficient) was also defined.

#### Participants and pharmacokinetic results

Twelve volunteers participated in this research, namely: 92% women and 8% men, age 28.82 ± 10.68 years, 1.60 ± 0.10 m in height (mean ± SD). Six groups of samples were collected for naproxen alone and six groups of samples for naproxen associated with esomeprazole.

**Fig 2:**
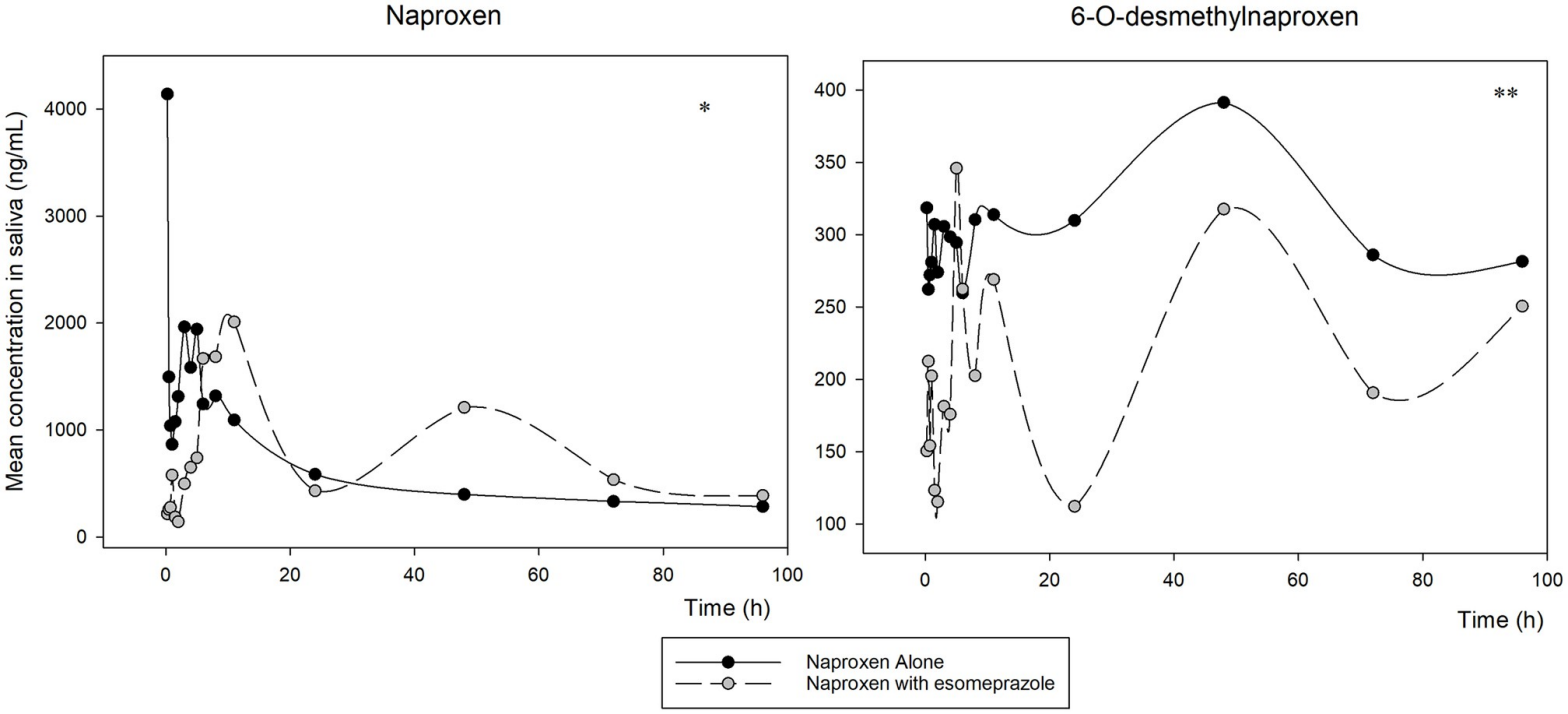
Mean of the concentrations found in the analyzed samples of naproxen (left) and 6-O-desmethylnaproxen (right) for the six volunteers who consumed the naproxen alone compared to naproxen associated with esomeprazole. Statistically significant difference *p<0.05 and **p<0.001.

**Fig 3:**
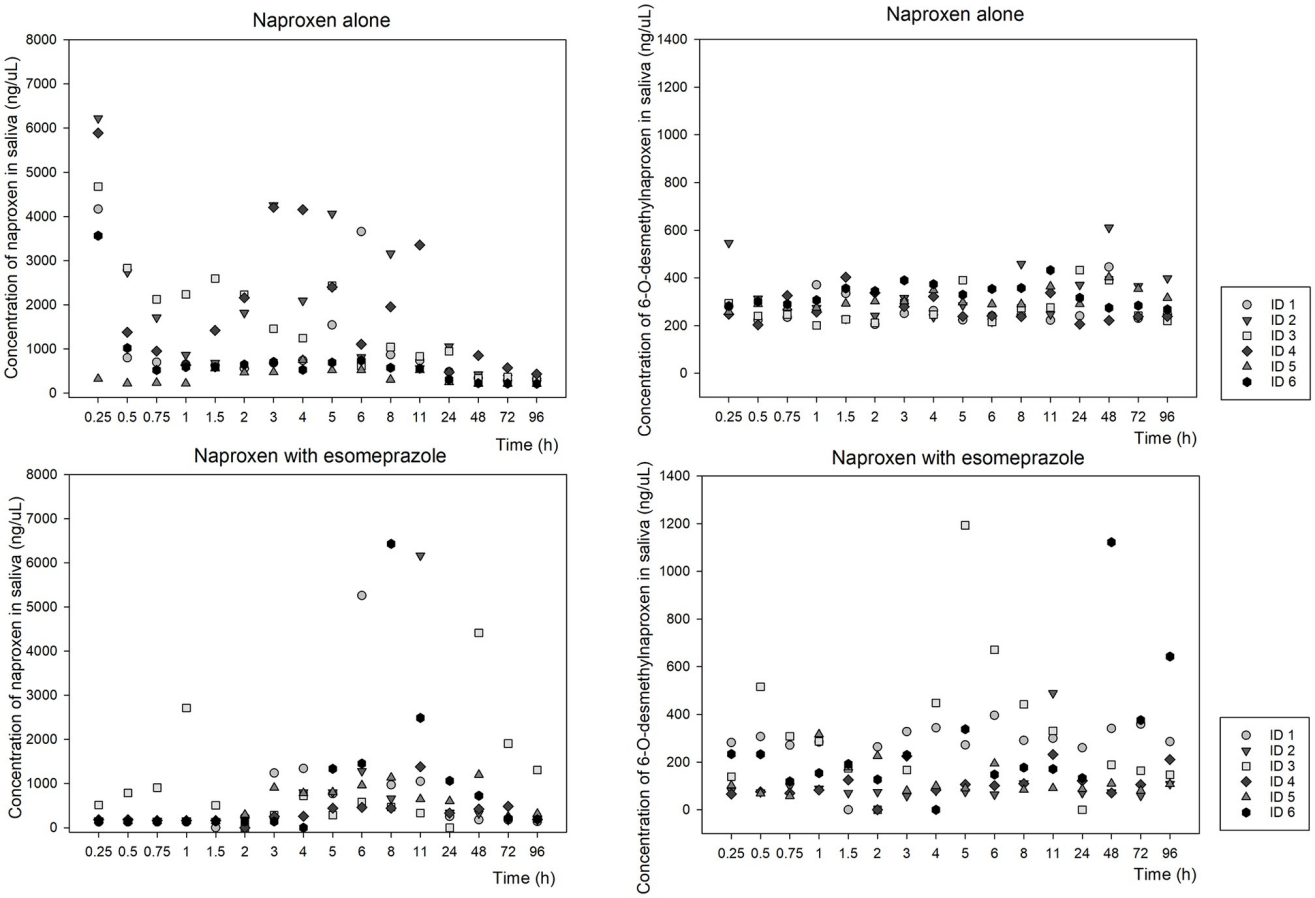
Graph representing the dispersion of each volunteer at each time point after taking both medications for naproxen (left) and its main metabolite (right).

With the average concentrations found at each time point after ingestion of the tablet of naproxen alone and associated with esomeprazole, it was possible to construct the following graphs (Fig 2), which gives us an overview of the behavior of naproxen metabolism (left) in 6-O-desmethylnaproxen (right) over time. Scatter plots for each volunteer at each time point after taking both medications are also plotted for naproxen (Fig 3—left) and its main metabolite (Fig 3—right).

Table 2 show the pharmacokinetic parameters obtained from the naproxen and its major metabolite, 6-O-desmethylnaproxen concentrations found in the analyzed saliva samples.

**Table 2:** Pharmacokinetic data of naproxen and 6-O-desmethylnaproxen for naproxen alone and associated with esomeprazole, provided by Phoenix WinNonlin software (version 8.1) from concentrations resulting from LC MS/MS analysis of saliva samples (n = 6 for each group).

**PK parameters**	**Naproxen**		**p value**
	**alone**	**with esomeprazole**	
**normal distribution**	**mean ± SD**		
Cmax (ug/mL)	4.60±2.50	2.04±0.78*	0.038
Vd/F (L)	0.56±0.29	0.26±0.17	0.054
**not normal distribuition**	**median (Q1—Q3)**		
Tmax (h)	0.17 (0.13–1.95)	13.18 (10.12–27.15)*	0.002
AUC0-t (h*ug/mL)	2.97 (1.82–7.87)	82.06 (51.90–157.00)*	0.002
Cl/F (ug/h)	7,29 (3.17–16.23)	0.01 (0.005–0.01)*	0.002
Kel (1/h)	6.11 (0.72–7.84)	8.29 (4.95–16.96)	0.240
T1_/2_ (h)	0.12 (0.09–1.35)	9.16 (7.16–41.40)*	0.002
**PK parameters**	**6-O-desmethylnaproxen**		**p value**
	**alone**	**with esomeprazole**	
**normal distribution**	**mean ± SD**		
Tmax (h)	15.82±13.66	19.5±20.06	0.718
Cmax (ug/mL)	0.480±0.10	0.34±0.18	0.133
Vd/F (L)	1.16±0.36	2.07±1.81	0.258
Cl/F (ug/h)	0.39±0.38	0.65±0.53	0.348
T1_/2_ (h)	10.96±9.47	13.51±13.90	0.718
**not normal distribuition**	**median (Q1—Q3)**		
AUC0-t (h*ug/mL)	20.62 (2.93–43.49)	9.48 (0.96–50.45)	0.818
Kel (1/h)	0.11 (0.04–0.35)	0.06 (0.34–0.80)	0.818

T_max_ e C_max_: time and value of the maximum observed concentration, respectively; AUC_0-t_: area under concentration versus time curve from the first observed concentration to the last one; Vd/F: estimated volume of distribution in total AUC; Clt/F: full clearance; Kel: elimination rate constant estimated from the regression line representing the terminal phase of the concentration-time profile; T_1/2_: terminal half-life of the drug.

* statistically significant difference, p<0.05.

**Fig 4:**
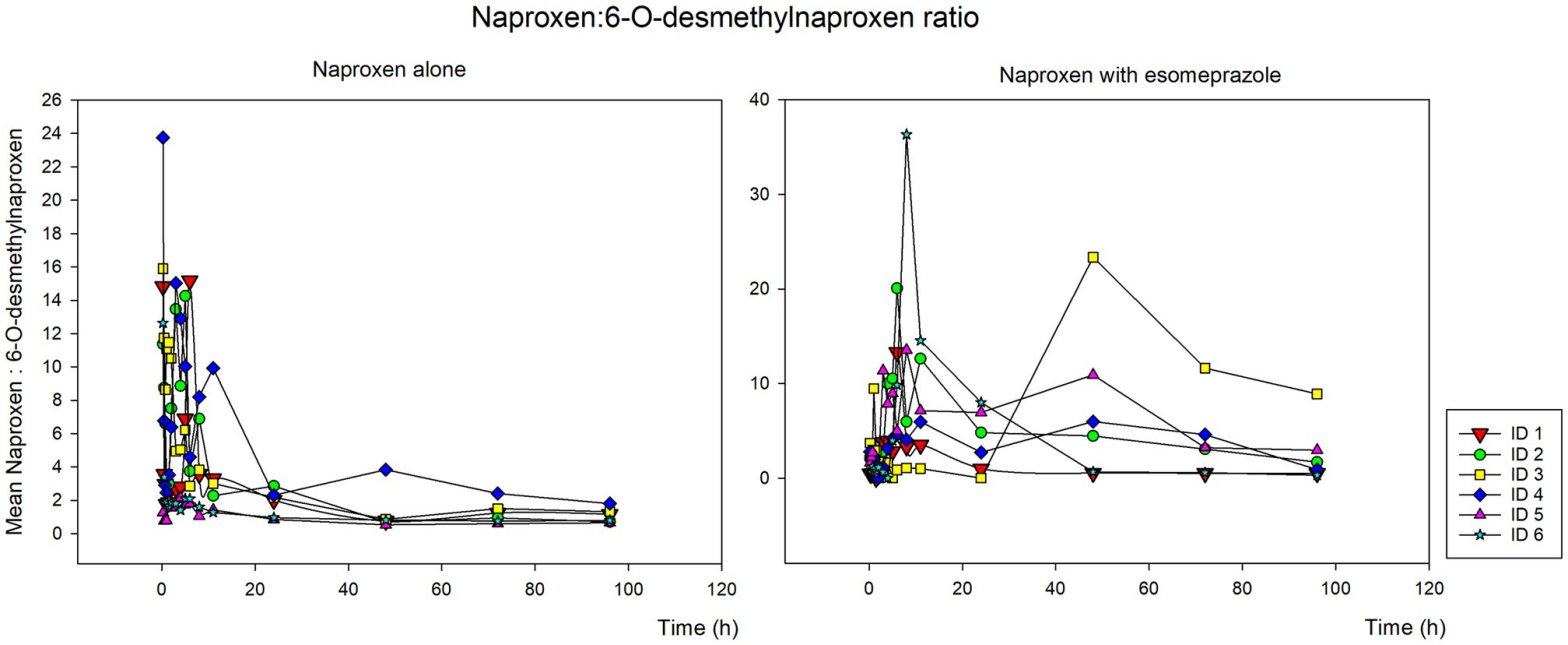
Ratio of concentrations found in saliva samples of naproxen in relation to the 6-O-desmethylnaproxen metabolite in the 6 volunteers who received the isolated naproxen tablet (500 mg–left) and naproxen with esomeprazole (500 + 20 mg–right), in relation to time.

To represent drug consumption and metabolite formation, the naproxen/6-O-desmethylnaproxen ratio was calculated in saliva samples in relation to the collection time, being graphically represented in Fig 4 (isolated naproxen–left—and naproxen associated with esomeprazole—right) [2, 3]. We can thus observe the metabolization time in a visual way in the two formulations analyzed.

### Analytical validation

Matrix effect of all saliva samples (n = 6) was absent (on line resource) for both naproxen and its major metabolite, 6-O-desmethylnaproxen, the coefficient of variation of the internal standard (IS) normalized matrix factor achieved for each analyte was less than 15% (Table 3) [2, 4].

**Table 3:** Concentrations of naproxen and 6-O-desmethylnaproxen saliva samples (5 samples).

	Naproxen		6-O-desmethylnaproxen	
	Concentration (ng / mL)	IS normalized matrix factor (CV)	Concentration (ng / mL)	IS normalized matrix factor (CV)
**Saliva** (6 samples)				
LQC	9.8	6.6	9.8	11.6
HQC	625	5.5	625	8.4

IS: internal standard; CV: coefficient of variation [(standard deviation / mean)] x 100.

All validation extraction methods of naproxen and its main metabolite, 6-O-desmethylnaproxen, in saliva samples are reported in Table 4. Briefly, linearity was 2.4 to 1250 ng/mL for naproxen and its major metabolite, 6-O-desmethylnaproxen, r2 = 0.992 for naproxen, r2 = 0.999 for its major metabolite, 6-O-desmethylnaproxen. The precision and accuracy assessments obtained a coefficient of variation lower than 15%, indicating a good degree of accuracy and reproducibility of the analysis performed. Intra-assay and inter-assay precision and accuracy represented by the coefficient of variation and the relative error, respectively, were less than 15% for naproxen and its major metabolite, 6-O-desmethylnaproxen in saliva samples.

**Table 4:** Validation parameters performed for analysis of naproxen and 6-O-desmethylnaproxen in saliva samples by LC-MS / MS.

Naproxen		6-O-desmethylnaproxen	
Linearity	2.4–1250 ng/mL		
*r* ^2^	0.992	0.999	
Equation of the line	f(x) = 3063.16*x+3572,39	f(x) = 750.715*x+5583.27	
Limit of quantification (ng / mL)	2.4	2.4	
Precision (CV %; n = 10)	12.5	13.6	
Accuracy (%)	3	11.1	
Intra-assay precision (CV %, *n* = 5)			
LLOQ (2.4 ng/mL)	12.4	LLOQ (2.4 ng/mL)	8.5
LQC (9.8 ng/mL)	10.7	LQC (9.8 ng/mL)	15.2
MQC (312.5 ng/mL)	5.3	MQC (312.5 ng/mL)	11.6
HQC (625 ng/mL)	8.2	HQC (625 ng/mL)	6.6
DQC (2500 ng/mL; 1:5)	8.3	DQC (2500 ng/mL; 1:5)	9.2
Interassay precision (CV %, *n* = 8)			
LLOQ (2.4 ng/mL)	13.6	LLOQ (2.4 ng/mL)	10.8
LQC (9.8 ng/mL)	11.7	LQC (9.8 ng/mL)	10.9
MQC (312.5 ng/mL)	14.1	MQC (312.5 ng/mL)	9.3
HQC (625 ng/mL)	6.7	HQC (625 ng/mL)	7.8
Intra-assay accuracy (RE %, *n* = 5)			
LLOQ (2.4 ng/mL)	-2.6	LLOQ (2.4 ng/mL)	9.3
LQC (9.8 ng/mL)	0.1	LQC (9.8 ng/mL)	-0.3
MQC (312.5 ng/mL)	13.6	MQC (312.5 ng/mL)	5.9
HQC (625 ng/mL)	-0.1	HQC (625 ng/mL)	3.7
DQC (2500 ng/mL; 1:5)	-8.9	DQC (2500 ng/mL; 1:5)	-10.5
Interassay accuracy (RE %, *n* = 8)			
LLOQ (2.4 ng/mL)	9.9	LLOQ (2.4 ng/mL)	10.6
LQC (9.8 ng/mL)	0.5	LQC (9.8 ng/mL)	0.4
MQC (312.5 ng/mL)	8.9	MQC (312.5 ng/mL)	5.1
HQC (625 ng/mL)	1.5	HQC (625 ng/mL)	5.6
Stability (RE %, *n* = 4)			
Short-term stability (12 h at 23°C)			
LQC (9.8 ng/mL)	7.4	LQC (9.8 ng/mL)	4.5
HQC (625 ng/mL)	14.1	HQC (625 ng/mL)	8.8
Post-processing stability (12 h at 4°C)			
LQC (9.8 ng/mL)	-6.8	LQC (9.8 ng/mL)	-8.8
HQC (625 ng/mL)	14.5	HQC (625 ng/mL)	11.9
Freeze / thaw cycle stability (−70°C)			
LQC (9.8 ng/mL)	-11.7	LQC (9.8 ng/mL)	-11.2
HQC (625 ng/mL)	-12.7	HQC (625 ng/mL)	-9.3

CV, coefficient of variation [(standard deviation / mean) x 100]; r, linear correlation coefficient, RE, relative error = [(observed concentration − nominal concentration)/nominal concentration] × 100; LLOQ, lower limit of quantification; LQC, low quality control; MQC, medium quality control; HQC, high quality control; DQC, quality control for dilution integrity.

After three freeze (-70°C) / thaw (23°C) cycles, naproxen and its major metabolite, 6-O-desmethylnaproxen maintained stable in saliva after 12 h at 23°C and processing samples up to 12 h at 4°C. These deviations were less than 15%.

The quality control to achieve dilution integrity (DQC– 2500 ng/mL–dilution 1:5) of naproxen and its major metabolite, 6-O-desmethylnaproxen had coefficient of variations lower than 15%.

## Discussion

Naproxen is extensively metabolized in the liver by the cytochrome P450 (CYP) system, CYP2C9 and CYP1A2, to 6-0-desmethylnaproxen. Neither parent drug nor metabolites induce metabolizing enzymes. Both naproxen and 6-0-desmethylnaproxen are further metabolized to their respective metabolite conjugates with acylglucuronide [5]. Considering that naproxen is a drug that is highly protein bound (~99%), like most NSAIDs, we can observe that the proportionality of drug and metabolite concentrations found in each volunteer was maintained (on line resource), showing that the use of saliva samples for this experiment was successful [1, 2, 6–8]. The high adherence of volunteers, in addition to the ease of collecting saliva, encourages us to increasingly use this proposed methodology.

In Fig 2, we can see that the metabolization in the association with esomeprazole is delayed in relation to the naproxen tablet alone, as expected by the delay in absorption, due to the presence of the proton pump inhibitor. It is worth remembering at this point that such formulation, in addition to the chemical properties of each component, has a particularity in relation to its content, where esomeprazole is found in a more external layer, being dissolved, and, therefore, absorbed at first, followed by of naproxen, which is found inside the tablet. Such physical difference may explain the great difference in the initial peak concentration of naproxen when it comes to the isolated tablet, as well as in the initial production and levels found of its main metabolite.

In Table 2 we can see that when naproxen is associated with esomeprazole, the time of maximum concentration is significantly altered, as well as maximum observed concentration, the area under the curve and half-life. In contrast, we see a decreased total clearance when we have the association with esomeprazole. The manufacturer of this combination itself—VIMOVO (trade name of the association of naproxen with esomeprazole) (AstraZeneca; London, United Kingdom) explicitly indicates the use of this medication for chronic and non-acute pain, stating that “VIMOVO is not recommended for the initial treatment of acute pain because the absorption of naproxen is delayed compared to other naproxen-containing products.” But it also reports that: “esomeprazole is rapidly absorbed with peak plasma concentrations reached in, on average, 0.43 to 1.2 hours”. [5, 9]. The results found in our research are corroborated here.

We know that patients who are chronically using NSAIDs for the treatment of rheumatological diseases can use them for 3 to 8 weeks in a row and can last for several months, and the association with proton pump inhibitors, such as esomeprazole, significantly improves adverse effects, gastric problems that can occur [5]. However, such an association was also effective in controlling acute pain after lower third molar surgeries in a study carried out in our laboratory [9]. If we take into account that the peak of inflammation occurs around 24 to 48 hours after the surgical trauma, with our experiments we can observe that both drugs would reach the maximum concentration within this period, but the association of naproxen with esomeprazole would take considerably longer time for that to happen [T_max_ 0.17 h (interquartile range, 0.13–1.95) for naproxen alone and 13.18*h (interquartile range, 10.12–27.15) for naproxen with esomeprazole, p = 0.002].

In the drug label itself, we see that the elimination half-life of naproxen is ~14 hours, regardless of the chemical form or formulation. In our work, where we see the free form of the drug present in saliva, which will effectively have the anti-inflammatory and side effects of the drug, we observed different values for both naproxen alone and associated with esomeprazole [T_1/2_ 0.12 h (interquartile range, 0.09–1.35) for naproxen alone and 9.16*h (interquartile range, 7.16–41.40) for naproxen with esomeprazole, p = 0.002].

As stated above, it is known that naproxen is extensively metabolized to 6-0-desmethylnaproxen in the liver, and this was the main motivation for the detection of this metabolite in our study. As described in its package insert, approximately 95% of the naproxen dose is excreted in the urine, primarily as naproxen (less than 1%), 6-0-desmethyl naproxen (less than 1%) or its conjugates (66–92%). About 3% of the dose or less is excreted in the feces. The rate of excretion of metabolites and conjugates is very close to the rate of elimination of naproxen from plasma. In saliva, we can observe very similar elimination values for both naproxen [K_el_ 6.11 1/h (interquartile range, 0.72–7.84) and 8.29 1/h (interquartile range, 4.95–16.96)] and its main metabolite (K_el_ 0.11 1/h (interquartile range, 0.04–0.35) and 0.06 1/h (interquartile range, 0.34–0.80)] both after taking the naproxen tablet alone and the tablet plus esomeprazole, respectively.

As with the concentrations of naproxen in saliva samples, the concentrations of its main metabolite, 6-O-desmethylnaproxen, are extremely low in this fluid (~1.9 μg/mL of naproxen and ~0.5 μg/mL of 6-O-desmethylnaproxen), demonstrating the need to use LC MS/MS for its detection [1, 2, 6, 7]. Also showing that the extraction methodology used in this part of the research was quite effective.

With the graphs generated in our study, it was possible to observe a much more variable naproxen:6-O-desmethylnaproxen ratio when naproxen is associated with esomeprazole (Fig 4), possibly due to the delay in absorption that the presence of the proton pump inhibitor can cause. Even with values well increased above 24 hours after taking the tablet.

In research involving pharmacokinetic and pharmacogenetic studies of NSAIDs, mainly those metabolized by the CYP [10, 11], the metabolite/drug ratio is an important tool for analyzing the altered results of mutated and non-mutated individuals in a determinate polymorphism [2, 10]. The main polymorphism that we can focus on when studying NSAIDs is that of CYP2C9, of great importance in the metabolic pathway, even when studying naproxen and its main metabolite 6-O-desmethylnaproxen. All pathways from metabolism, elimination and secondary pathways, as well as possible drug interactions, must be considered in studies of this type [3].

The association of naproxen with esomeprazole showed increased values of maximum concentration time and area under the curve in drug concentrations in relation to the naproxen tablet alone, probably, such differences are due to the delay in the absorption of naproxen when it is associated with the drug proton pump inhibitor, esomeprazole. As well as reduced values of total clearance when naproxen is combined with esomeprazole. Such differences were not noticed in relation to its main metabolite, 6-O-desmethylnaproxen, which obtained similar values in all PK parameters. Studies with a larger number of volunteers should confirm these results.

When we observe the concentrations found at each time after the ingestion of the naproxen tablet associated with esomeprazole, the delay in this absorption and consequently in the metabolization of the drug is clear, which could greatly interfere with the action in a situation of acute pain, where we want an action faster pain relief, but if we consider the peak of inflammation, which occurs 24 to 48 hours after the surgical trauma, we can use it without restrictions, since the levels approach in this period.

The analysis of pharmacokinetic parameters in saliva samples proved to be a viable and less invasive option with good results and analysis of PK parameters. The analysis of concentrations by LC MS/MS is extremely necessary because NSAIDs, including naproxen and its main metabolite, 6-O-desmethylnaproxen, are drugs highly bound to the protein, therefore found in very low levels in saliva samples.

It was possible to quantify both naproxen and its main metabolite, 6-O-desmethylnaproxen, in saliva samples using LC-MS/MS after an oral dose of 500 mg of naproxen alone or associated with esomeprazole (500 mg + 20 mg) in volunteers in both situations. The LC-MS/MS methodology developed in this research proved to be fast, simple, sensitive, and of high performance for each drug and allowed us to analyze its pharmacokinetic parameters, in both situations, associated or not with esomeprazole.

## Supporting information

S1 Checklist CONSORT 2010 checklist of information to include when reporting a randomised trial*. (DOC)
